# Biomimicry of the Hawk Moth, *Manduca sexta* (L.), Produces an Improved Flapping-Wing Mechanism

**DOI:** 10.3390/biomimetics5020025

**Published:** 2020-06-04

**Authors:** Kenneth Moses, Mark Willis, Roger Quinn

**Affiliations:** 1 Department of Mechanical and Aerospace Engineering, Case Western Reserve University, Cleveland, OH 44106, USA; roger.quinn@case.edu; 2 Department of Biology, Case Western Reserve University, Cleveland, OH 44106, USA; mark.willis@case.edu

**Keywords:** flapping-wing micro aerial vehicle (FWMAV), flapping-wing mechanism (FWM), power measurement, lift measurement, *Manduca sexta*, mechanical efficiency

## Abstract

Flapping-wing micro air vehicles (FWMAVs) that mimic the flight capabilities of insects have been sought for decades. Core to the vehicle’s flight capabilities is the mechanism that drives the wings to produce thrust and lift. This article describes a newly designed flapping-wing mechanism (FWM) inspired by the North American hawk moth, *Manduca sexta*. Moreover, the hardware, software, and experimental testing methods developed to measure the efficiency of insect-scale flapping-wing systems (i.e., the lift produced per unit of input power) are detailed. The new FWM weighs 1.2 grams without an actuator and wings attached, and its maximum dimensions are 21 × 24 × 11 mm. This FWM requires 402 mW of power to operate, amounting to a 48% power reduction when compared to a previous version. In addition, it generates 1.3 gram-force of lift at a flapping frequency of 21.6 Hz. Results show progress, but they have not yet met the power efficiency of the naturally occurring *Manduca sexta*. Plans to improve the technique for measuring efficiency are discussed as well as strategies to more closely mimic the efficiency of the *Manduca sexta*-inspired FWM.

## 1. Introduction

In recent years, there has been increased usage of miniature aircraft controlled remotely with semi-autonomous capabilities. These aircraft, colloquially referred to as “drones”, have a multitude of applications including aerial photography and videography, short-range communication, meteorology, transportation and delivery, surveillance and reconnaissance, and search and rescue. These many applications, in addition to improvements in ease of use and a reduction in cost, have generated an increase in demand for these vehicles.

Smaller drones are of particular interest due to their potential for increased agility and mobility in smaller spaces and decreased visual detectability. A small drone with a wingspan of less than 15 cm and weighing less than 50 g is sometimes referred to as a micro aerial vehicle (MAV); a term established by the Defense Advanced Research Projects Agency (DARPA) in 1997 [1]. Current MAVs suffer from a number of shortcomings or performance barriers that need to be overcome. Increasing flight duration, range, durability, and safety while decreasing noise are important topics in advancing the development of MAVs. Researchers are seeking novel solutions to address these problems, and many have turned toward a biologically inspired approach. In nature, flight forces are generally achieved through flapping wings. In contrast, engineered aircraft most commonly utilize continuous rotary motion with a propeller or rotor to generate lift.

Animals with the capability of sustained flight are found in three categories: The class Insecta (insects) within the arthropod phylum, the class Aves (birds) within the Chordata phylum, and the order Chiroptera (bats) within the class Mammalia of the Chordata phylum. All have served as models for the technological development of unmanned aerial vehicles. Advances in manufacturing of small-scale devices have enabled a shift towards smaller MAVs that take advantage of improved electromechanical power densities and power-weight efficiencies. Additionally, complexities in the wing structures and wing trajectories of birds and bats have proven to be challenging to replicate mechanically. Insects, however, have passive wing structures with no muscles or joints that actively actuate the exterior of the thorax. Insect-scale drones fit into the specifications of MAVs and have the potential to address several of the performance barriers previously discussed. It is for these reasons that we are basing our work on a flying insect.

For reference, our paper entitled “Artificial *Manduca sexta* forewings for flapping-wing micro aerial vehicles: How wing structure affects performance” provides background information relevant to the work presented here and is recommended as a precursor [2].

### 1.1. Flapping-Wing MAVs, Systems, and Mechanisms

Every successful flapping-wing micro aerial vehicle (FWMAV) must have a set of components that produces the flight forces necessary for sustained flight. This set of components, referred to here as the flapping-wing system, is often comprised of one or more actuators, a flapping-wing mechanism that converts the motion of the actuator(s) into the desired motion of the wings, and the wings themselves.

The performance of flapping-wing MAVs, their systems, and mechanisms can each be evaluated in a variety of ways. FWMAVs are most often characterized by overall aerodynamic lift for a given size and weight of the vehicle. For example, experiments on a bio-inspired flexible flapping-wing MAV show time-varying forces during the flapping period and the effect of wing layout and flexibility [3]. Methods that determine the performance of a range of FWMAV configurations and show lift for a given power consumption have been established [4,5,6]. Performance of component subsystems of a MAV, such as the flapping mechanism, has been difficult to quantify in bench tests, where overall performance of an FWMAV has been calculated in terms of lift produced as compared to natural weight of birds and insects [7].

Despite these challenges, researchers have been able to characterize flapping mechanism efficiencies using a few different types of methods. Four-bar flapping mechanisms are often used due to the ease of their modeling and fabrication, and characteristic low transmission loss. The essential mechanics of flapping mechanisms in the insect thorax have been modeled and tuned for aerodynamic performance with parameterized wings through perturbation analysis [8]. Torque generated by a flapping mechanism rocker, in a crank-rocker four-bar mechanism, derived from strain gauge measurements has been used to determine the optimum motor reduction gear and lengths of the mechanism for a dove-size MAV [9]. In another instance, a four-bar mechanism was used to minimize transmission loss for a dragonfly-size MAV [10]. Furthermore, mechanism transmission momentum was modeled for a beetle-size scotch yoke mechanism [11], and the overall performance of the wing and flapping mechanism was determined by using a strain gauge to measure lift.

**Figure 1:**
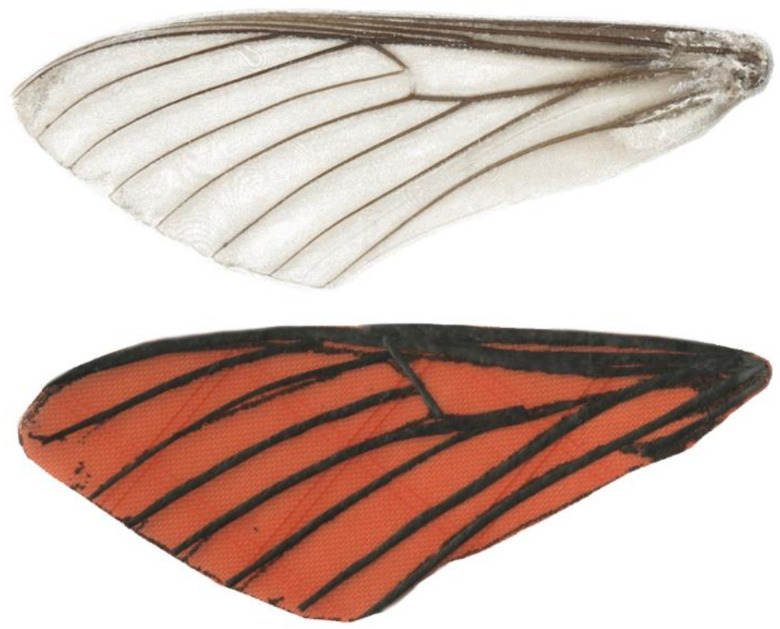
(**top**) 2D scan of natural forewing excised from *Manduca sexta* (**bottom**). Artificial forewing used in this study. These manufactured wings have an approximate wing length of 50 mm, chord length of 20 mm, and mass of 40 mg. Their membrane is made of Icarex™ ripstop polyester, and their venation structure is constructed of Gurit prepreg unidirectional carbon fiber [2].

The complexity of wing design and mechanism design, in addition to the inherent coupling between the two, suggests that it may be useful to separate the problem into two research areas. The design of biomimetic wings involves optimizing size, structure, weight, and flexibility to be similar to animal wings, while the design of flapping mechanisms involves optimizing mechanical devices to achieve performance density similar to the animal’s muscles and mechanisms. The assessment of flapping-wing systems at the hawk moth scale is challenging because unlike rotary-wing-powered craft, useful force must result from a continuous repetitive cycle of wing acceleration and deceleration. The approach we take is to keep the wing design fixed and to evaluate flapping mechanisms by measuring overall performance of the coupled wing-flapping system. We are using this approach for two reasons: we have previously published a method for fabricating moth-like wings that we used for this study, and this approach simplifies the process (Figure 1) [2].

### 1.2. Efficiency as a Performance Metric

The amount of lift generated per unit of power input to a flapping-wing system is an essential performance metric in assessing the efficiency of a flapping-wing mechanism. The more lift that the wings produce while maintaining a constant power draw indicates greater performance and fewer energy losses in the system. If we assume the size and weight of the power plant remains the same, a reduction in energy losses results in longer flight durations and thus an increase in range. Alternatively, depending on the desired flight characteristics, agility or payload capacity could improve if the power plant is reduced in size and weight. Consequently, many of the performance metrics of MAVs can benefit from examining the performance of individual components of a MAV and lead to improved overall MAV efficiency.

In addition to advancing many of the common characteristics of MAVs, the power required to produce enough lift for flight for a given flapping-wing mechanism defines the criteria necessary for proper actuator selection. Once the power requirements are determined, the actuator type and size can be chosen followed by other MAV components such as energy storage.

Furthermore, efficiency of MAVs and their subsystems is a metric often found in literature and used to compare alternate designs as well as theoretical limits. Flapping-wing system overall efficiency must consider wing design and mechanism design together. Their performance is closely coupled. Wing design is dependent on the design of the mechanism and vice versa. For example, a FWMAV with moth-like wings should flap its wings in a moth-like trajectory and frequency.

### 1.3. The Hawk Moth Manduca Sexta

We narrow our focus towards replicating larger animals within the class of insects as it affords us additional engineering design space. The larger insects are arguably easier to replicate since their components themselves are larger and can be recreated with a variety of traditional and conventional manufacturing techniques such as subtractive and additive manufacturing. Another benefit to focusing on larger flying insects is that as we scale up within this class of arthropods, they are capable of more payload, which is useful in some FWMAV applications.

Analysis of power requirements and losses in actuators and transmission mechanisms suggest a crossover between use of piezoelectric actuators at the fruit fly scale to use of rotary motor actuators at the hummingbird scale and larger [12]. For nano-scale FWMAVs smaller than 1 mm, weighing less than 1 g and with a flapping frequency greater than 100 Hz, non-spinning (linear type) actuators such as piezoelectric or thermal actuators have suitable power efficiencies [13]. Micro-scale solutions typically involve motor-driven crank mechanisms [14].

**Figure 2:**
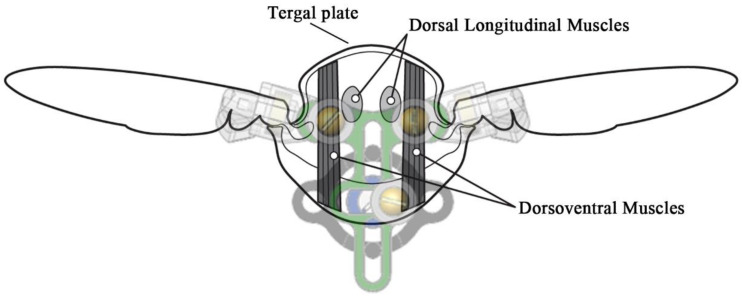
A cross section of an insect’s thorax depicting the indirect flight muscle system. Our latest flapping-wing mechanism is overlain, indicating similarities between the thorax and the mechanism. The Scotch-yoke of the mechanism encompasses both the dorsal longitudinal muscles (DLMs) and dorsoventral muscles (DVMs) as it drives the wings in both directions [23].

The hawk moth *Manduca sexta* is one of the most attractive model organisms for FWMAV development because of its ability to hover in gusty conditions, its size for operating in confined areas, and its weight relative to payload capacity. *Manduca sexta* is one of the largest flying insects. The wings and thorax are the key functional elements of any flying insect and are of particular interest in mimicking for FWMAVs. Researchers have analyzed and quantified the flight characteristics of *Manduca sexta* [15,16,17,18,19]. Hawk moth wings and thoraces have been studied to provide detailed structure and performance data. The hawk moth’s flapping mechanism incorporates an indirect flight muscle system where the muscles in the thorax act on the exoskeleton to flap its wings (Figure 2). Reverse engineering this structure led to the idea that the muscle energy transferred to the wings could be characterized by measuring the energy needed to compress the insect’s thorax. Studies have determined that the elastic modulus of the tergum (tergal plate) is approximately 5.02 ± 0.31 GPa and that the large flight muscles need to produce 31.4 ± 2.6 mW of mechanical power to flap the wings [15,20]. Particular focus on measuring and characterizing thorax muscle power output has produced experimental evidence and survey results to support robust models and estimates of the thorax power density [21]. Experiments involving mechanical measurements have determined the average mechanical power required to produce hawk moth upstroke and downstroke flapping, and the power density of the hawk moth dorsal muscle groups [22]. Cranston and Palazotto (2014) summarized *Manduca sexta* specific power output and input, and the most recent estimates are 112 W/kg and 1170 W/kg, respectively [15].

Researchers have also constructed models of the animal and analyzed the performance of their reconstructions. Studies have produced finite element models of the hawk moth wings and thorax. A physical wing-body model of the hawk moth has been constructed to evaluate the aerodynamic performance, derive input power requirements, and evaluate performance improvements associated with wing flexibility. Findings indicate flexible wing mechanisms enhance overall efficiency even though they require more input power [24]. Notable general findings reveal that the inertial load associated with wing acceleration and deceleration can require as much as half of the total power input [25]. These measurements and models of hawk moth thorax power densities serve as goals for evaluating performance of functionally equivalent mechanical flapping mechanisms.

## 2. Methods

### 2.1. Flapping-Wing Mechanism 2.1 Development Methods

This team previously presented two flapping-wing mechanism (FWM) designs [2,26,27]. These mechanisms were developed by applying first principles of physics, implementing state-of-the-art practices in the field of insect biomimicry, and through design iteration by qualitative observation of the mechanism performance and inspection of *Manduca sexta* in flight. Furthermore, initial creation and advancement of the mechanism benefited from investigations of the capabilities of the manufacturing process and the mechanical properties of the material in use. In addition, a common approach to progress performance of mechanical components is to address areas of weakness and points of failure. For instance, in a dynamical model such as this, focusing on regions of high stress, unexpected or abnormal amounts of friction, or locations of poor tolerance where undesired movement occurs can prove vital to meeting the expectations of the design. In the case of biomimicry, the design specifications are determined by the model organism, *Manduca sexta*. More specifically, we are attempting to meet or exceed the animal’s dynamics including its size, shape, mass, efficiency (power-to-lift ratio), and wing motion. By continuing to employ this approach, we were able to make more improvements to the design (i.e., develop a mechanism that more closely mimics *Manduca sexta*).

### 2.2. Flapping-Wing Mechanism Efficiency Measurement Methods

To determine the efficiency of flapping-wing mechanisms, we measured five variables: load voltage, load current, mechanism position, flapping frequency, and lift generation. Efficiency was calculated from a ratio of input power consumption to output power generation. Input power was derived from estimates of load voltage and load current. Output power was derived from measurements of lift. The position and flapping frequency were also measured to discern component contributions and evaluate model compliance with the expected performance characteristics of the hawk moth. The flapping-wing mechanism was run at specific duty cycles, and the measurements were recorded. A range of duty cycles were used to sweep through a range of flapping frequencies. Flapping frequencies were not controlled with feedback; they were dependent on the mechanism configuration.

**Figure 3:**
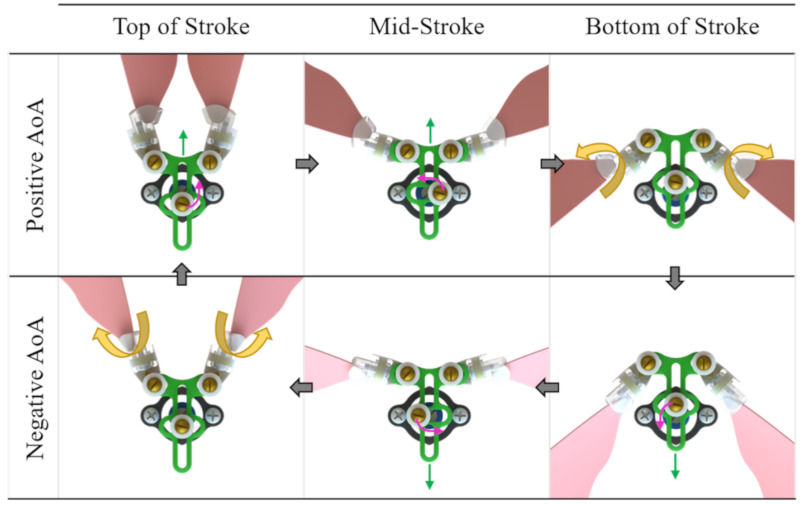
Complete flapping cycle of FWM 2.1 as viewed from the top. Starting from the top left corner of the diagram, the wings begin at the top of their stroke with positive angle of attack (AoA). As we proceed to the top middle picture, the crank (blue) rotates in the counter-clockwise direction, thus driving the yoke (green) upward and the wings (pink) downward. Subsequently, the crank rotates another quarter of a turn and the wings reach the bottom of their stroke. At this point, the wings passively flip from a positive to a negative angle of attack (note that this does not occur instantaneously like depicted in the diagram but over a brief amount of time and after the crank has rotated some small amount). The crank continues to rotate, driving the yoke downward and the wings upward. Lastly, with the wings at the top of the stroke, aerodynamic and inertial forces cause them to flip (passively rotate along the spanwise axis) into a positive angle of attack. Like the previous wing flip, this motion also does not occur instantaneously but over a short amount of time and after the crank has rotated a small amount.

The design of the modified scotch-yoke flapping-wing mechanism converts continuous rotary motion into oscillatory flapping motion (Figure 3). A 6 V 10:1 high-power carbon brushed dual-shaft Pololu micro metal gearmotor was selected to drive the flapping-wing mechanism. This DC motor has a maximum speed of 3000 RPM (50 Hz), maximum output power of 1.3 watts at 6 volts, specific output power of 136.8 W/kg, a size of 10 × 12 × 26 mm, and a mass of 9.5 g. These specifications meet or exceed the specifications of what is known about the animal with the exception of mass. Meeting mass specifications for the actuator was not deemed essential at this stage of development because we are focused on the mechanism and its performance, not the entire system. A number of other factors also contributed to the selection of this particular motor. For example, its accessibility, reliability, ease of control, cost, performance history, and familiarity were all considered when deciding to use this electric motor.

Mechanism power input was recorded quickly and accurately. In its simplest form, instantaneous electrical power is a function of load current and load voltage. Since a 20 kHz PWM signal was used to control the electric motor, the load current and load voltage had to be sampled at a minimum 40 kHz to guarantee that both the highs and lows of the signal were captured; otherwise, a bias could occur. After procedures were refined and recording software incrementally improved, we were able to record power measurements at a rate of 50,000 samples per second with a no-load standard deviation of 0.32 mW.

A high-speed position sensor was integrated into the system so that the mechanism position and flapping frequency could be recorded. The lift was expected to vary throughout the wingbeat. Thus, it was important to know the position of the mechanism and wings at the time lift and power were measured. Additionally, by recording the mechanism position, data could be extracted over the course of one wingbeat and processed to assess the mechanism’s cyclical performance. Flapping frequency could also be calculated by taking the time derivative of the mechanism position. The microcontroller was configured to capture velocity values every millisecond (1000 samples per second) and record the time at each indexer pulse. A full wingbeat cycle was interpolated from the indexer pulses. The gearbox on the motor has a ratio of (35 × 37)/(13 × 10): 1, or approximately 9.96: 1. Therefore, the indexer pulses occur roughly 10 times every cycle.

**Figure 4:**
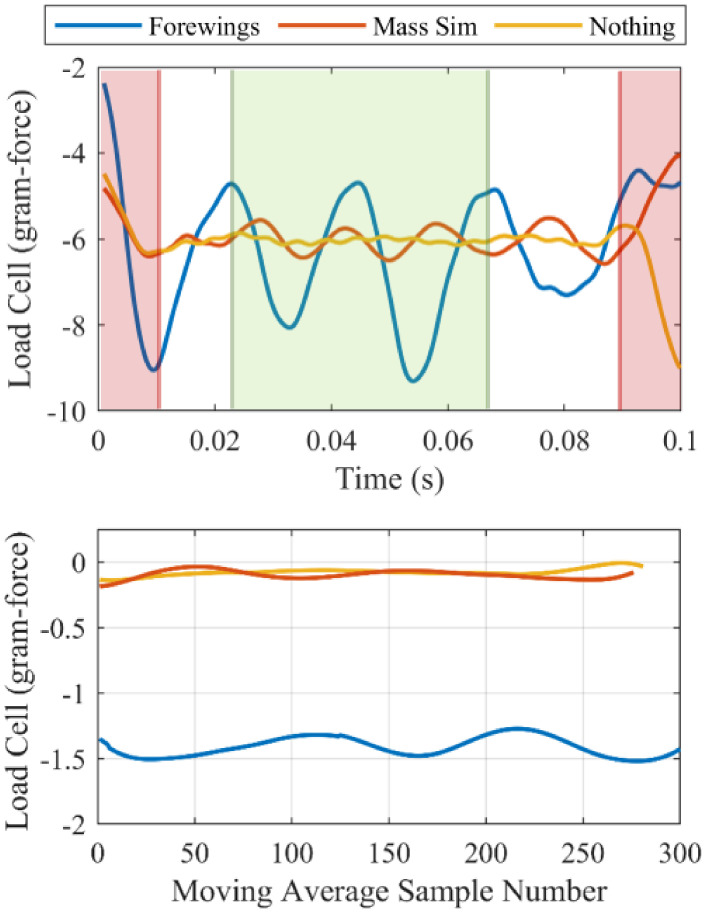
(**top**) Filtered data from one trial of the flapping-wing system in each of the three configurations: forewings attached (blue), mass simulators attached (red), and nothing attached (yellow). Highlighted in red are the data subjected to edge effects from filtering and discarded. Highlighted in green is an example of the region of interest (one wingbeat of data) that is averaged to produce one moving average sample, shown in (**bottom**).

Lift was measured with a micro load cell and a 32-bit analog-to-digital converter. Samples were captured at a rate of 7200 Hz. For each experiment trial, load cell data for more than one wingbeat were captured. This affords the ability to calculate a variable moving average across the duration of the data based on the most recent quadrature encoder information. The timing data recorded from the quadrature encoder indexer were used to interpolate the amount that the motor rotated (i.e., the incremental location of mechanism within the wingbeat) in terms of number of wingbeats for each load cell data point captured. A leading moving average was applied to the filtered load cell data where the window size corresponds to a full wingbeat as determined by the interpolated position. Once the moving average was applied, the first and last 10% of the data was discarded in order to remove the edge effects caused by filtering. This procedure (Figure 4) resulted in a number of estimates for the lift generated per experiment trial. Lastly, these estimates were averaged and compared to other mechanism configurations.

The Tiva C microcontroller has 256 KB of flash memory. At the sampling rates and sizes of the recorded data, a maximum of 100 milliseconds could be captured by the microcontroller. Considering a minimum of one full wingbeat was needed to assess the performance of the mechanism, 100 milliseconds of data allows for flapping frequencies no less than 10 Hz. *Manduca sexta* flaps its wings at approximately 25 Hz, well above the minimum of 10 Hz, translating into 2.5 wingbeats worth of data. If it is desirable to record additional wingbeats or lower flapping frequencies, external memory could be added. All events and sampling rates are shown in the event timing table (Table 1).

**Figure 5:**
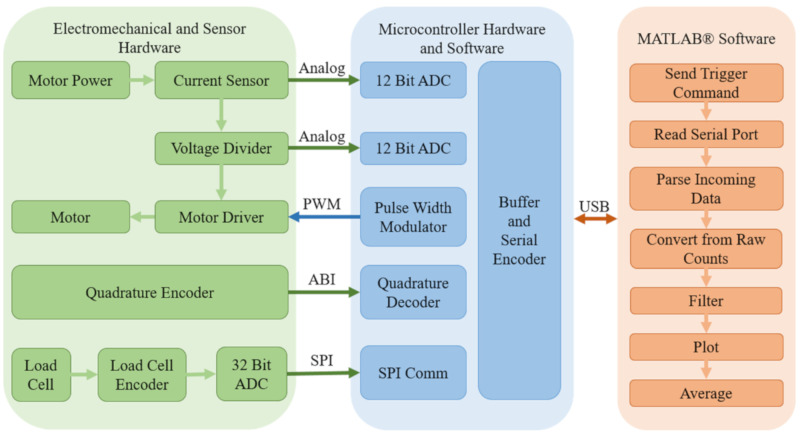
Complete system diagram. The system is comprised of three subsystems: electromechanical and sensor hardware, microcontroller hardware and software, and MATLAB^®^ software. Two analog signals and four digital signals are used to communicate between subsystems. Pulse width modulation (PWM), ABI quadrature encoder, serial peripheral interface (SPI), and universal serial bus (USB) are the four digital communication protocols used.

Figure 5 presents a diagram containing all hardware and software components as well as communication protocols used to obtain the desired performance metrics of the flapping-wing mechanisms in question. For more details on the hardware components and their configurations, see Appendix A.

### 2.3. Experimental Methods

Three experiments were contrived and conducted to analyze the performance of the newly developed FWM and to compare it to other FWMs in literature as well as fabricated by this team. These experiments are the Motor Load Calibration and Efficiency experiment, the System Load Comparison experiment, and the System Lift Production experiment. The Motor Load Calibration and Efficiency experiment established the relationship between motor power consumption and motor load and can be found in Appendix B. The two other experiments are elaborated below.

An experiment trial consisted of start-up time, data capture, and stopping time. A MATLAB^®^ program sent a command through the PC’s serial port to trigger the experiment trial sequence. The command indicated a desired pulse-width-modulated duty cycle (25–100%), a 5 s time delay before data were recorded to allow the flapping-wing mechanism to reach a steady state condition, and the type of trial conducted (step input). Once the trial was completed, all data that were stored in the buffer on the microcontroller were transmitted to the PC via the serial port. MATLAB read and stored the incoming data and then performed all post-processing operations such as conversion from raw counts, resampling, and filtering.

#### 2.3.1. System Load Comparison

**Figure 6:**
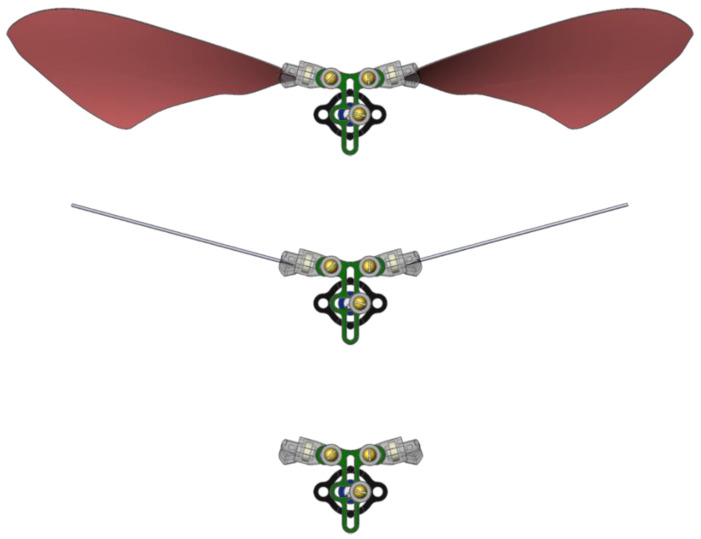
(**top**) FWM 2.1 with artificial forewings attached. (**middle**) FWM 2.1 with metal rods attached that are equivalent to the mass of the forewings but have minimal air resistance. (**bottom**) FWM 2.1 with nothing attached.

The most recently developed FWM was compared to its predecessor in its ability to convert continuous rotary motor motion into oscillatory flapping-wing motion. The power consumptions of FWM 2.0 and FWM 2.1 were measured by performing trials at seven duty cycles from 25–100% in increments of 12.5%. Comparing power consumption of FWM 1.0 was not necessary because FWM 2.0 already exhibited significant improvements. FWM 2.0 and FWM 2.1 were tested in three different configurations: with artificial forewings attached, with forewing mass simulators attached, and without anything attached (Figure 6). Making measurements with these configurations allows the effects of forewing aerodynamic forces and forewing inertial forces to be isolated and observed independently of one another. The forewing mass simulators were thin spring steel bars (0.508 mm diameter, 38 mm length, 0.06 g) intended to replicate the inertia of the forewings but had little cross-sectional area and thus produced minimal aerodynamic forces.

#### 2.3.2. System Lift Production

FWM 2.1 was tested in the three configurations (with forewings attached, with mass simulators attached, and with nothing attached) for its ability to produce lift (Figure 6). For each configuration, five experiment trials were conducted at duty cycles that have similar flapping frequencies (approximately 20 Hz). Power consumption, lift generation, and flapping frequency were all recorded for durations of 100 milliseconds, corresponding to two wingbeats of data at 20 Hz. Additionally, two wing orientations were tested and their results are presented in Section 3.3. In order to compare performance results, the lift production was estimated by averaging the load cell measurements over the span of one wingbeat as explained in Section 2.2 and seen in Figure 4.

## 3. Results

### 3.1. Flapping-Wing Mechanism 2.1

Through the implementation of the development methods discussed in Section 2.1, the flapping-wing mechanism (FWM) has been improved and more closely mimics *Manduca sexta* in size and mass. All major components were reduced in size and mass with the motor casing having the largest reduction of 94.6% by mass (Table 2).

**Figure 7:**
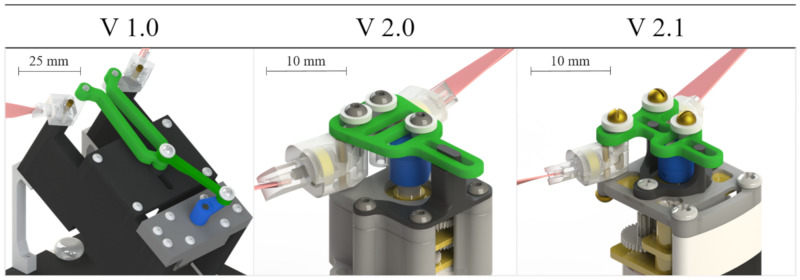
Progression of flapping-wing mechanism (FWM) development from left to right. In all versions shown, the forewings (pink) are attached to a wing adapter (clear) and shoulder joint (clear), which are driven by a linkage (green) between the shoulder joints and motor crank arm (blue). In versions 2.0 and 2.1, a ball bearing (yellow) is housed within the shoulder joint to allow minimal friction during spanwise rotation of the forewings. The shoulder joints rotate around small axles (brass) to achieve the oscillatory flapping motion. The motor is held in place via the motor casing, (gray) and stators (black) are used to guide the linkages through their desired motion.

FWM 2.1 is lighter, more compact, more robust, and more precise and efficient (as shown in Section 3.3) in its flapping motion. It incorporates several changes to the mechanism relative to FWM 2.0. First, a smaller ball bearing (outer diameter decreased from 4.76 mm to 3.18 mm) was incorporated into the shoulder joint (Figure 7), resulting in a shorter motor shaft and a corresponding lower profile. Second, the stator was changed from a single-sided prismatic joint to a double-sided revolute joint. This improves the directionality of the yoke, ensuring that the yoke maintains a linear path as it is driven by the crank arm and greatly reduces the amount of contact surface area between components, thereby lessening friction within the mechanism. Hardware screws were changed from stainless steel size 0–80 to brass size 00–90. Decreasing the size of the motor casing involved using two inverted 00–90 screws partially milled to their cores as axles for the shoulder joints. Furthermore, we were able to remove additional motor casing material by using two M1.6 × 3 mm screws and securing the mechanism directly to the gearbox rather than making a clasp around the entire gearbox as was previously done. Profile reduction required removing a large portion of the motor shaft, drilling and tapping into the motor shaft (along its major axis), and securing the crank arm to the motor shaft with a 00–90 × 1/8″ flat head screw. With this method, we were able to reduce the length of the crank arm from 0.09″ to 0.063″, resulting in geometrical changes in subsequent linkages for FWM 2.1 and allowing for room for the double-sided revolute joint design seen in Figure 7.

### 3.2. Motor Load Calibration and Efficiency

**Figure 8:**
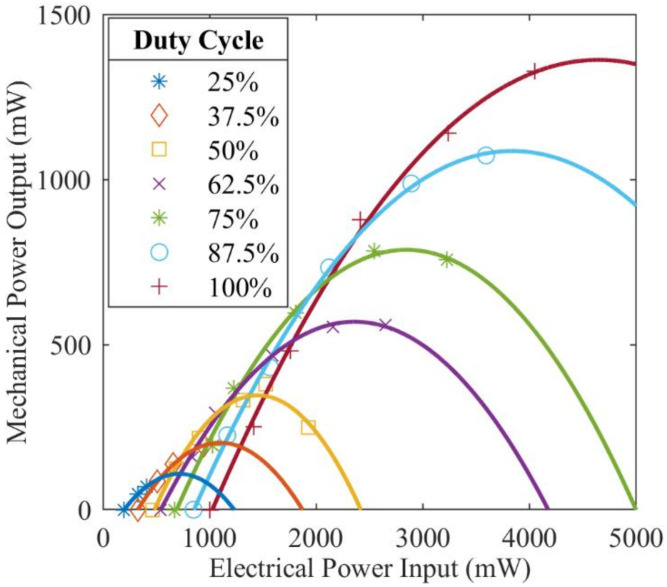
Mechanical power output as a function of electrical power input from measured data of seven duty cycles. Each duty cycle’s data set is fitted with a second-order polynomial, all of which have a minimum correlation coefficient of 0.978. These polynomials were used to convert measured electrical power consumption into mechanical power output when the motor is subjected to unknown loads. Although not included in this diagram, the factory specifications for no-load and stall conditions support the measured data and the resulting trend lines.

The Motor Load Calibration and Efficiency experiment (discussed in Appendix B) produced second-order polynomials used to convert the measured electrical power input to the electric motor into values that represent the mechanical power output from the motor (Figure 8). Data from this experiment run at full duty cycle confirmed anticipated current, speed, efficiency, and power output curves with r-squared correlation values greater than 0.985. The maximum observed efficiency throughout this experiment was 34.03% and occurred at a speed of 2171 RPM and a torque load of 5.01 N∙mm. The maximum mechanical power output measured was 1327 mW.

### 3.3. System Load Comparison

Table 3 summarizes the results from the load comparison experiment. This table presents data post-conversion from measured electrical powered input to the motor to mechanical power output from the motor (Figure 8). In all three configurations, the advancements made with FWM 2.1 vs. FWM 2.0 reduced the necessary mechanical power output from the electric motor (i.e., mechanical power input to FWM) by at least 15%. When comparing the performance of the flapping-wing mechanism without the forewings or forewing mass simulators attached, power reductions of 70.5% and above were observed. For the configuration with the forewings attached to the mechanism and thus a complete FWM system, necessary mechanical power output was reduced by a minimum of 24.9% to upwards of 53.2%, occurring at 87.5% duty cycle and 25.0% duty cycle, respectively.

The results from Table 3 and their respective flapping frequencies for each duty cycle were fitted with a second-order polynomial and interpolated at the flapping frequency of 24.7 Hz (approximate frequency of the animal). Analyzing the full system with forewings attached, the new flapping wing mechanism (FWM 2.1) required 48.6% less mechanical power from the electric motor than the old flapping wing mechanism (FWM 2.0) at the animal’s flapping frequency. Throughout the span of all duty cycles, FWM 2.1 with forewings attached reduced power consumption by an average of 33.2%.

### 3.4. System Lift Production

System lift production results (lift generated, power input measured, and flapping frequency observed) for five trials of FWM 2.1 in three configurations are shown (Table 4). These results indicate that, in the configuration with nothing attached and the configuration with the mass simulators attached, a negligible amount of lift was detected, as expected.

## 4. Discussion

### 4.1. Measurement Hardware and Procedures

The measurement procedures mentioned within this manuscript provide detailed descriptions on how a low-cost, high-precision, and high-accuracy testbed was developed for determining power consumption and lift generation of insect-scale FWMs. The procedures produce consistent results with low standard deviations when averaged across five or more trials. However, it should be possible to obtain more consistency with lower standard deviations through faster sampling rates, higher signal-to-noise ratios, faster communication speeds, and longer trial durations (limited by the amount of memory onboard the microcontroller). For example, an alternate configuration for the position sensor provides potential for improving wing position estimates. The AMS AS5147P high-speed position sensor was configured for ABI communication, which replicates an incremental encoder with an indexer. A higher-bandwidth and more precise option would be to utilize the sensor’s built-in Serial Peripheral Interface (SPI) communication protocol, which provides absolute position measurements. This should result in a more accurate estimation of the timestamps and number of data samples of one wingbeat, thereby providing better approximations of the average lift generated across the entire wingbeat.

The Phidgets micro load cell performed well in our tests. In its calibration, we observed linear, repeatable results, with a small amount of hysteresis that was accounted for in the experiments. We were able to achieve load cell measurements accurate to ±30 mg force, sampling at 7200 Hz. While this was sufficient to detect discrepancies in lift measurements between configurations, we would like to achieve measurement accuracy of ±1 mg force. As such, areas of improvement include vibration isolation, increased measurement precision and accuracy, and additional degrees of freedom. Vibration frequencies greater than the flapping frequency are irrelevant, make it difficult to discern the lift data results, and should be discarded. This work utilizes a low-pass filter to remove the high-frequency vibration observed in the load cell measurements. However, there are alternatives to consider, such as mechanical dampening or more robust filtering. For example, a linkage to decouple the diametric magnet from the extended motor shaft and only transmit torque was conceived but not implemented. Lastly, additional degrees of freedom would help to observe moments and forces vital to determining flapping wing system stability.

Electrical power measurements achieved results accurate to less than ± 1 mW in best-case scenarios but varied up to ±200 mW under large loads and at high motor speeds. Two options that may reduce these variations are switching to a digital current sensor (from Texas Instruments INA169 to INA219) and using a brushless motor instead of a 6 V brushed DC motor. The data showed that much of the noise in voltage and current measurements occurred because of spiking (presumed to be from the brushes) as well as the 20 kHz Pulse Width Modulated (PWM) signal used to control the speed of the motor. A brushless motor configuration would likely remedy this.

In summary, this work resulted in measurement hardware and procedures that are capable of capturing power draw and lift generation with enough accuracy and at a high enough sampling rate to observe and identify performance specifications of insect-scale FWMs. This much-needed capability paves the way for thorough analysis of future design iterations of FWMs in which geometrical configurations, material selections, and hardware component selections can be optimized based on empirical data.

### 4.2. Experiment Results

The load calibration procedure resulted in a maximum efficiency of 34% for the electric motor and motor controller. Determining the curve fit for each duty cycle (Figure 8) permits isolation of the power produced by the motor from the motor controller. The efficiency losses of the motor controller and motor are accounted for when converting from electrical power input to mechanical power output. Maximum mechanical power output is consistent with the manufacturer’s listed specifications at approximately 1.3 W. Additional load experiments were considered in order to determine the motor’s transient response by running experiments with various angular moments of inertia attached to the motor’s shaft. This could prove useful in developing accurate motor simulations but was not necessary to obtain initial performance data on the FWMs.

Comparing the equivalent mechanical power output of the electric motor (i.e., power input to the FWM) required by FWM 2.0 to FWM 2.1 yielded positive results with FWM power input reductions observed in all FWM 2.1 configurations. In particular, when the FWMs were run without anything attached, reductions of 71% to 97% were observed in FWM 2.1. At the animal’s flapping frequency of 24.7 Hz power reductions of 82%, 22%, and 48% were calculated for FWM 2.1 by itself, FWM 2.1 with forewing mass simulators attached, and FWM 2.1 with forewings attached, respectively (see Section 3.2 for information regarding how these values were calculated). Furthermore, at the same frequency and with FWM 2.1, the power required was measured to be 24 mW, 161 mW, and 402 mW, respectively. Therefore, we can conclude that the power required to overcome aerodynamic forces is 1.7 times that of the power required to overcome inertial forces with FWM 2.1.

**Figure 9:**
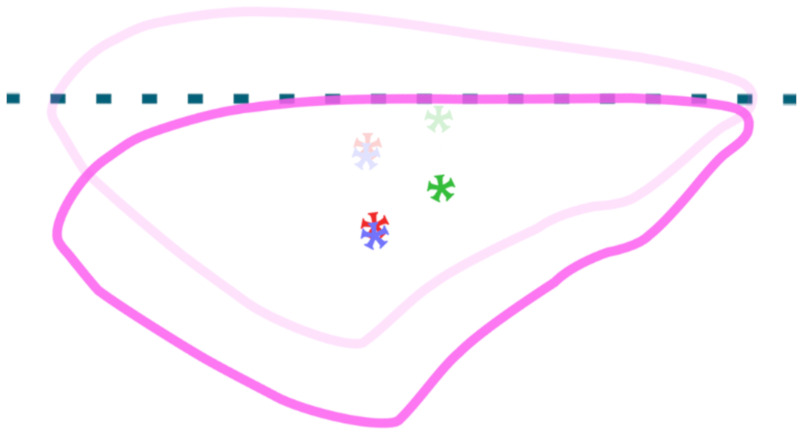
The original orientation (semi-transparent) and new orientation (opaque) of the forewings are shown. Points of interest associated with the forewing are included; center of mass (green), area centroid (red), and center of pressure (blue). In the new orientation, all three points of interest have moved farther from the flip axis (dashed line) to improve passive pronation and supination of the forewing.

Initial experiments involving the FWMs with forewings attached showed insignificant amounts of lift generated. Upon reviewing high-speed video of both the flapping wing mechanism with artificial forewings and the animal, it was determined that the forewings’ rotations were slow compared to the animal’s, lagging substantially behind the time of stroke reversal within the wingbeats. Modifications to the forewing adapters were needed in order to enable them to rotate faster and earlier within the downstroke and upstroke of the wingbeat cycle. The forewing adapters were originally designed for the flip axis of the forewings to pass through the maximum wing length. However, it was thought (and later confirmed through observation of the animal) that the flip axis resides along the leading edge of the wing (Figure 9). The original orientation of the forewing with respect to the spanwise rotation prohibited passive rotation, thereby preventing lift production. This change in forewing orientation drastically improved the lift production.

Lift production over the course of a trial period varied greatly (Figure 4). It was necessary to develop a technique to estimate the length of the wingbeat and calculate the average lift generated over the wingbeat in order to obtain a number representative of the flapping wing system’s performance. Ultimately, a lift force of approximately 1.3 g force was calculated at a wingbeat frequency of 21.6 Hz. *Manduca sexta* hawk moths typically weigh between 1–2 g; therefore, we have measured enough lift to hover smaller hawk moths at a lower flapping frequency. One can expect that if the flapping frequency were increased to 24.7 Hz, even more lift would be produced. However, flapping at a specific frequency would require implementing a control algorithm, which could affect power estimates.

The advancement from FWM 2.0 to FWM 2.1 is substantial in our findings. However, in comparison to the power output estimates of *Manduca sexta* found in literature, the latest flapping wing system (FWM 2.1 with forewings) is still less efficient. For instance, Cranston and Palazotto reported body-mass-specific power output densities between 33–54 mW/g [15]. Assuming an average body mass of 1–2 g, this corresponds to a power output of 33–108 mW [28]. FWM 2.1 outputs 361 mW of mechanical power while producing 1.3 g force of lift. Additionally, using the same approach, power input is estimated to be 191–936 mW based on literature, whereas the power input of FWM 2.1 with forewings is measured to be 1260 mW. These results suggest that the inefficiencies of the current flapping wing system lie within the conversion of the forewing motion into lift.

The findings presented in this paper are representative of FWM 2.0 and 2.1 in combination with the *Manduca sexta*-mimicked artificial wings used in the experiments. They may serve as a benchmark in evaluation of combined flapping mechanisms and forewing configurations. The *Manduca sexta* forewings and FWM interaction is closely coupled, involving dynamic load feedback within the wingbeat cycle that would be difficult to simulate on independent components. However, flapping mechanism subsystem designs can be individually evaluated through measuring the performance of the coupled system. Additionally, modifications to the current FWM, forewing alterations, and new flapping-wing systems can be analyzed with the hardware, software, and experimental procedures discussed.

## 5. Conclusions

The need for agile MAVs with long flight durations and extended range is well established. One potential solution is to implement an FWM inspired by *Manduca sexta* as they have been shown to be highly efficient in hovering and extremely agile in their flight maneuvers. Researchers have taken different approaches towards developing insect-inspired FWMAVs and have considered, and analyzed, a variety of FWMs.

This manuscript describes a portion of an ongoing project with the goal of mimicking the hawk moth *Manduca sexta* in hopes of achieving similar flight performance. Previous work described the development and analysis of artificial forewings and suggested that the forewings developed would produce comparable amounts of lift if used in an FWMAV [2]. To advance the design and fabrication process of the artificial forewings and the FWM that drives the wings, improvements to the existing testbed for measuring flapping-wing system efficiency were needed. The hardware, software, and measurement procedures used in data collection of the flapping-wing system are now capable of power measurements with less than ±1 mW standard deviation at a sampling rate of 50 kHz, and lift measurements with less than ±30 mg-force standard deviation at a sampling rate of 7200 Hz. This was accomplished by calibrating the voltage, current, and lift sensors and performing a controlled applied torque experiment that established the relationship between electrical power input and mechanical power output of the electromechanical components. We were able to achieve high resolution and accuracy with our testing apparatus even though it was entirely constructed from inexpensive commercially available components.

The most recent iteration of artificial forewings attached to the latest design version FWM produced 1.3 g force of lift while consuming 1.26 W of electrical power and flapping at a wingbeat frequency of 21.6 Hz. This FWM weighs just 1.2 g without the actuator, power source, and forewings attached. In our experiments, it is shown to require 82% less power than the previous FWM at the animal’s average wingbeat of 24.7 Hz to convert continuous rotary motion from the electric motor into oscillatory flapping-wing motion. The entire flapping-wing system (the actuator, FWM, and artificial forewings) requires 48% less power than the flapping-wing system with the prior FWM. The new FWM accomplishes this level of performance because of improved placement of the forewing adapter for faster passive spanwise forewing rotation and the implementation of a newly designed smaller yoke within the scotch-yoke mechanism.

While these outcomes indicate substantial improvement from previous designs, there is still work to be done. In comparing the power requirements of the current FWM to those of *Manduca sexta*, we see that the FWM demands more power to achieve similar amounts of lift. Mechanical power output demand of the FWM was measured to be 361 mW, whereas the animal is estimated to use 33–108 mW. We need to further optimize the design for better efficiency with the goal of reaching numbers closer to what is reported for the animal. One way that this could be accomplished is by incorporating a form of energy storage into the drive train that activates during stroke reversal [29]. Additionally, literature proposes that torsional springs be introduced into the forewing pronation and supination, resulting in greater lift production [30]. Once the desired efficiency of the FWM is reached, our focus should turn towards the electronics required to drive the flapping-wing mechanism (i.e., the microcontroller, battery pack, motor, and motor driver). The electronics implemented in our testing apparatus were chosen to accommodate the sensors needed to evaluate the performance of the flapping-wing system. A new suite of electronics that are smaller and lighter must be selected for the purpose of developing a flight-worthy FWMAV. These advancements could potentially allow for an FWMAV that realizes the flight characteristics of *Manduca sexta*.

## Figures and Tables

**Table 1:** Event timing table.

Sampling Rate (μs)	Frequency (Hz)	Event Type	Device	Comm. Protocol
20	50,000	Power (V × I)	2 × 12-bit Onboard ADC ^a^	Analog
50	20,000	Pulse Width Modulated Signal	Tiva C PWM Module	PWM
139	7200	Load Cell Acquisition	Protocentral 32-bit ADC	SPI
1000	1000	Motor Position	AMS AS5147P Position Sensor	ABI
40,000	25	Wing Beat	Flapping Wing Mechanism	USB
100,000	1	Sample Size	Tiva C Flash Memory	USB

^a^ (ADC) Analog to Digital Converter; (PWM) Pulse Width Modulated; (SPI) Serial Peripheral Interface; (USB) Universal Serial Bus.

**Table 2:** Specifications of each flapping-wing mechanism.

Version	1.0	2.0	2.1
Linkage Type	Crank-Slider	Prismatic Joint Scotch-Yoke	Scotch-Yoke
Total Mass ^a^ (grams)	110	6.2	1.2
Max. Dimensions (mm) (L × W × H)	76 × 76 × 64	33 × 33 × 21	21 × 24 × 11
Component Mass (grams)			
2 DOF Shoulder Joint	1.03	0.30	0.07
Wing Adapter	0.16	0.08	0.03
Linkage	2.12	0.18	0.08
Crank Arm	0.47	0.12	0.05
Stators	49.4	0.21	0.06
Motor Casing	9.55	2.91	0.16
Sum ^b^	62.7	3.79	0.45

^a^ Total mass and maximum dimensions of the mechanism without the motor and forewings. ^b^ Sum of the major components. Does not include additional hardware such as screws, ball bearings, or axles.

**Table 3:** Mechanical power input (mW) to FWM 2.0, FWM 2.1, and percentage reduced (R).

Duty Cycle	FWM Only			FWM w/ Simulated Masses			FWM w/ Forewings		
	v2.0	v2.1	(R)	v2.0	v2.1	(R)	v2.0	v2.1	(R)
25%	48 ± 9	9 ± 6	82%	34 ± 8	20 ± 6	42%	54 ± 8	26 ± 4	53%
37.5%	78 ± 16	10 ± 18	87%	78 ± 17	49 ± 18	37%	124 ± 19	89 ± 10	28%
50%	128 ± 32	4 ± 18	97%	140 ± 33	88 ± 24	37%	301 ± 22	207 ± 28	31%
62.5%	195 ± 17	58 ± 15	71%	270 ± 13	202 ± 29	25%	503 ± 17	334 ± 10	34%
75%	312 ± 30	65 ± 26	79%	424 ± 37	361 ± 45	15%	780 ± 4	540 ± 33	31%
87.5%	468 ± 33	91 ± 28	81%	644 ± 43	461 ± 40	28%	1061 ± 12	797 ± 37	25%
100%	808 ± 51	182 ± 13	78%	903 ± 25	704 ± 13	22%	1354 ± 6	940 ± 36	31%

**Table 4:** System lift production results of FWM 2.1.

FWM Configuration	Lift (mg Force)	Mechanical Power Input (mW)	Flapping Frequency (Hz)
FWM Only	91 ± 20	739 ± 145	19.9 ± 0.4
FWM w/Simulated Mass	84 ± 31	745 ± 156	19.7 ± 0.6
FWM w/Forewings	1299 ± 73	1261 ± 249	21.6 ± 0.7

**Figure A1:**
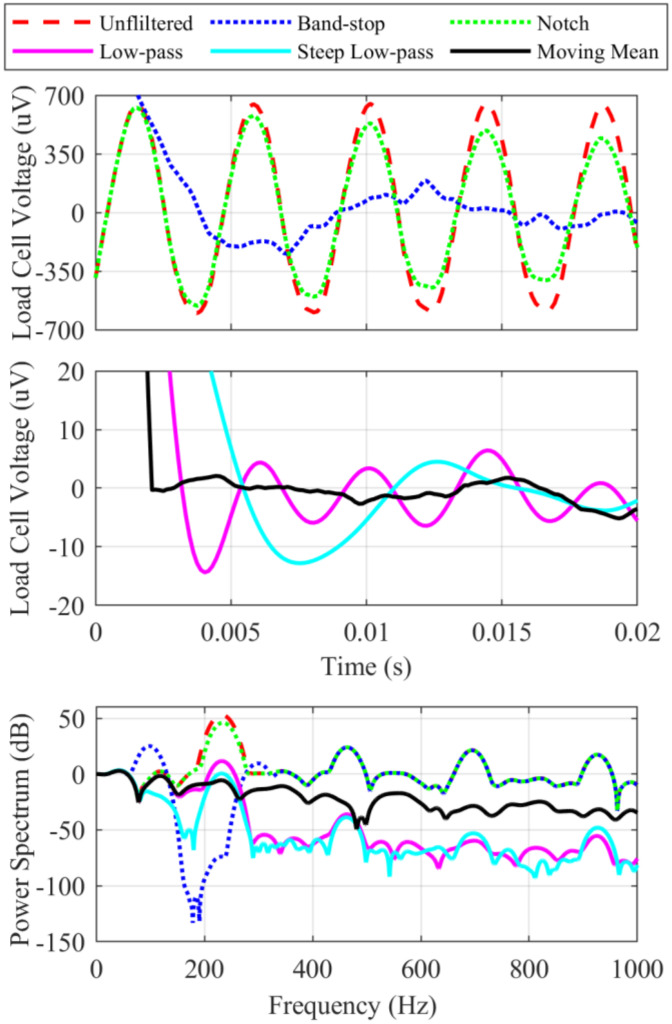
Filters designed to mitigate high-frequency noise. (Top) and (middle) have the same time scales; however, the low-pass, steep low-pass, and moving mean filters drastically reduce the amplitude of the signal and therefore require a smaller load cell voltage range for their results to be visible. (Bottom) The impact of the filters on the power spectrum.

**Figure A2:**
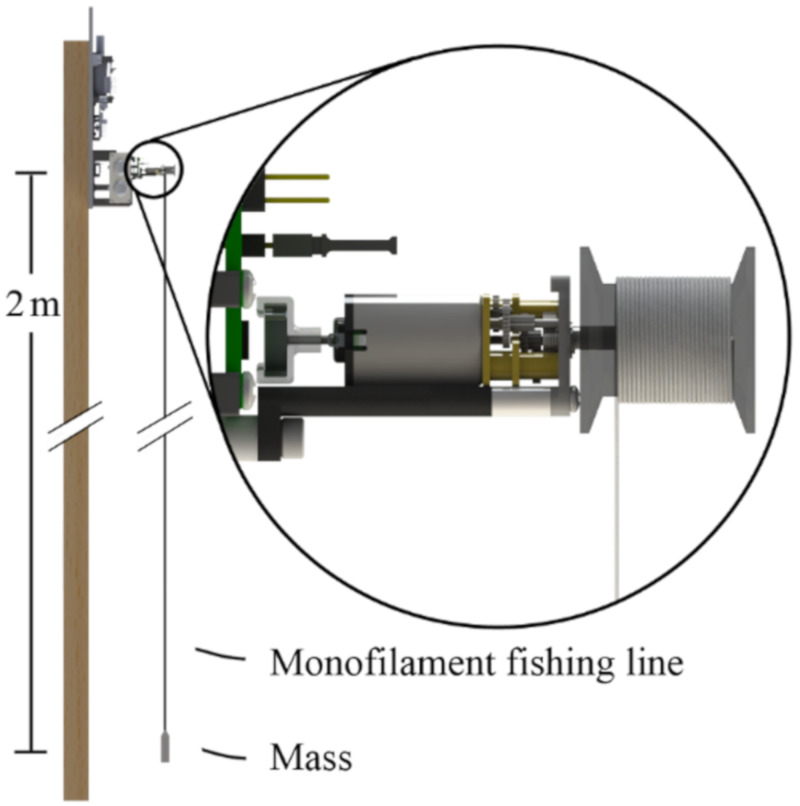
Depiction of motor load calibration experiment. Care was taken to ensure that the cord does not spool, thus preventing the radius from changing and maintaining a constant torque applied to the motor throughout the experiment.

**Figure A3:**
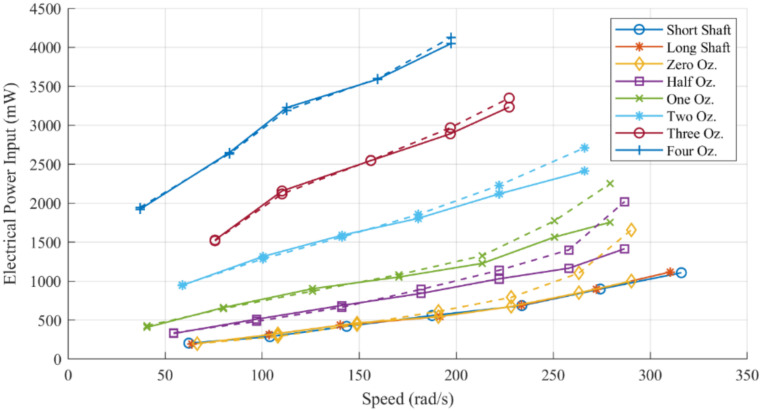
Electrical power input measurements at various duty cycles and load configurations with unadjusted (dashed line) and adjusted (solid line) data values shown. The adjusted values are determined by finding the electrical power input difference between the experiments with and without the pulley attached, interpolated at the speed of interest. This difference is then subtracted from the measurements of the weighted pulley experiments to provide the adjusted electrical power input. As expected, the adjusted values for the zero ounce measurement align with the short shaft and long shaft measurements. The impact of the pulley’s inertia on the measured power input is most noticeable at speeds above 160 rad/s (25.5 Hz). To accommodate both flapping-wing mechanisms, different gearboxes with the same gear ratios but unique shaft lengths were used. This test showed that the different gearboxes alone did not alter the load on the electric motor.

## Appendix A. Hardware Configuration Details

### Appendix A.1. Hardware Component List

**Table A1:** List of hardware components.

Part Name	Manufacturer	Part Number	Website/URL
Tiva™ C Series TM4C123G LaunchPad Microcontroller	Texas Instruments	EK-TM4C123GXL	http://www.ti.com/tool/EK-TM4C123GXL
Tattu LiPo Battery Pack 1300mAh 45C 3S 11.1V	Tattu	TA-45C-1300-3S-XT60	https://www.genstattu.com/tattu-1300mah-45c-3s1p-lipo-battery-pack-with-xt60-plug.html
eBoot LM2596 DC to DC Buck Converter	eBoot/Texas Instruments	LM2596S-ADJ	https://www.ti.com/product/LM2596
DRV8838 Single Brushed DC Motor Driver Carrier	Pololu/Texas Instruments	2990	https://www.pololu.com/product/2990
INA169 Analog DC Current Sensor Breakout	Adafruit/Texas Instruments	1164	https://www.adafruit.com/product/1164
AS5147P Position Sensor Adapter Board	AMS	AS5147P	https://ams.com/as5147padapterboard
Phidgets Micro Load Cell (0–100 g)—CZL639HD	Phidgets	3139_0	https://www.phidgets.com/?prodid=230
Protocentral ADS1262 32-bit Precision ADC Breakout Board	Protocentral/Texas Instruments	PC-4143	http://www.ti.com/lit/ds/symlink/ads1262.pdf

### Appendix A.2. Motor Power and Control

The motor was powered by an 11.1 V 3-cell lithium polymer battery that was stepped down to 6 V using an LM2596 buck converter step-down module. A 20 kHz Pulse Width Modulated (PWM) signal was used to control the DC motor. Two electrical components create the 20 kHz PWM signal: A Texas Instrument’s Tiva C Launchpad EK-TM4C123GXL microcontroller and a Texas Instrument’s DRV8838 motor driver. The microcontroller served many purposes, one of which was generating the PWM signal. The DRV8838 motor driver is an H-bridge that amplifies the PWM signal, making it suitable to drive the selected motor. See Figure 5 for a complete system diagram.

### Appendix A.3. Power Measurement

Power measurement was accomplished by using the Tiva C microcontroller’s onboard analog-to-digital converters (ADCs), a voltage divider comprised of two 10 kΩ resistors, and an Adafruit INA169 current sensor. The microcontroller’s ADCs have 12-bit resolution and an input voltage range of 0–3.3 V and are capable of up to 2 million samples per second with 180-degree phasing. The ADCs were configured to take four samples, each being four microseconds apart. This ensured adequate time in between samples for the current sensor to settle. In order to do this, the ADC clock was slowed to 250 kHz. The first sample of each channel was disregarded and the second sample of each channel recorded. The ADCs were configured to trigger their sequence of four samples at intervals of 20 microseconds (50 kHz sampling rate). MATLAB’s built-in low-pass filter was applied to the raw data from the ADCs with a passband frequency of 50 Hz (well above the target flapping frequency of 24.7 Hz), steepness of 0.85, and stopband attenuation of 60 dB, which is similar to approaches found in the literature [15]. The voltage divider was used to halve the load voltage to approximately 3.0 V, thereby falling within the input range of the ADCs. The sensitivity of the current sensor was modified to be 2.2 V per Amp by soldering a 22 kΩ load resistor to the breakout board. Therefore, at the electric motor’s stall current of 1.5 A, the current sensor output, the ADC full range value of 3.3 V.

### Appendix A.4. Position and Flapping Frequency Sensing

The AS5147P provides 12-bit resolution at a maximum speed of 28,000 RPM and sends angle position data through an ABI incremental encoder interface. A neodymium, 6 × 2.5 mm, diametric magnet is attached to the extended shaft of the electric motor. The Tiva C microcontroller has quadrature encoder interface (QEI) modules built in. The QEI modules integrate position over time, determine direction of rotation, and capture an estimate of the velocity of the encoder.

The micro load cell is a shear-type load cell with a rated output of 600 μV/V and supply voltage between 3–10 V DC. The Tiva C microcontroller communicates with the ADS1262 via SPI. The ADS1262 was configured for 7200 SPS with a Sinc4 filter and 32× gain.

### Appendix A.5. Measuring Lift Production

Lift was measured with a Phidgets micro load cell (0–100 g) and a Protocentral ADS1262 32-bit analog-to-digital converter capable of 38.4 kSPS with a 32 PGA. Preliminary testing of the devised lift measurement technique showed large oscillations in the raw lift values while the motor was running. The frequency of these oscillations was observed to coincide with the motor speed and not the flapping frequency (due to the gearbox, the motor rotates approximately 10 times faster than the flapping frequency). The cause of these oscillations was determined to be vibrations transmitted to the load cell from a slight eccentricity of the rotating diametric magnet and not the flapping motion of the wings nor an imbalance in the rotating armature of the electric motor as initially suspected. Post-processing the raw lift data by resampling and filtering the data achieved the necessary reduction in noise to observe the load cell signal. Data from the ADS1262 were captured as soon as they became available, and despite it being configured for 7200 Samples per Second (SPS), the data were not always precisely recorded every 138.8 microseconds. They were therefore resampled at 7200 Hz to reflect a fixed sampling rate. After testing band stop, low pass, and moving average filters, a low-pass filter was selected with a passband frequency of 30 Hz, steepness of 0.99, and stopband attenuation of 10 dB to remove the noise from the vibrations caused by eccentricity of the diametric magnet (Figure A1).

Linearity of the load cell was checked by applying multiple loads of varying amounts. As evident from the experiment, the r-squared value from linear regression of the data is extremely high (greater than 0.99). To account for drift and hysteresis in the load cell, a calibration sequence was performed prior to every experiment trial. The procedure was as follows; first, 700 samples were collected from the load cell without operating the mechanism. The average of these 700 samples establishes the baseline zero value (no lift) of the load cell. Second, a mass of 7291.2 milligrams was added on top of the mechanism to the load cell, and subsequently another 700 samples were recorded. The average of these samples along with the average of the no lift samples established the conversion between raw data counts from the ADS1262 to lift in units of milligrams. One more set of 700 samples was recorded without the additional mass and was used to reduce any hysteresis effects in the lift calculations.

## Appendix B. Motor Load Calibration and Efficiency

The Motor Load Calibration and Efficiency experiment established the relationship between motor power consumption and motor load (Figure A2). A constant load was applied to the electric motor by fastening a pulley (12.32 mm in diameter and 14 mm in length) to its shaft and suspending weights (0.5, 1, 2, 3, and 4 ounces) from the pulley with high-strength monofilament fishing line. A step input was sent to the motor with a programmed duty cycle, and power consumption data were recorded after allowing the motor to reach steady state (300 motor rotations). Mechanical output power was calculated from applied motor torque and RPM. Calibration tests were performed in seven configurations: the motor only, the motor with pulley attached and no mass suspended, and the motor with pulley and five different weights suspended. As a result, motor efficiency was assessed.

Results from the motor load calibration experiment indicate the electric motor’s ability to efficiently convert electrically stored energy into mechanical power output. Values were adjusted to account for the power required to rotate the pulley with no mass attached (Figure A3).
